# Role of Polyphenols from the Aqueous Extract of *Aloysia citrodora* in the Inhibition of Aflatoxin B1 Synthesis in *Aspergillus flavus*

**DOI:** 10.3390/molecules28135123

**Published:** 2023-06-30

**Authors:** Laura F. Cadenillas, Christopher Hernandez, Sylviane Bailly, Guillaume Billerach, Vanessa Durrieu, Jean-Denis Bailly

**Affiliations:** 1 Laboratoire de Chimie Agro-industrielle (LCA), Université de Toulouse, INRAE, INPT, 4 Allée Emile Monso, 31030 Toulouse, France; laura.cadenillas.s@gmail.com (L.F.C.); hernandezhernandezchristopher@gmail.com (C.H.); guillaume.billerach@toulouse-inp.fr (G.B.); vanessa.durrieu@ensiacet.fr (V.D.); 2 Mycoscopia, 3 rue Jean Monnet, 31470 Fonsorbes, France; mycoscopia@gmail.com; 3 UMR 1208 IATE Ingénierie des Agropolymères et Technologies Émergentes, INRAE, Institut Agro, Université de Montpellier, 2 Place Viala, 34060 Montpellier, France; 4 École Nationale Vétérinaire de Toulouse, 23 Chemin des Capelles, CEDEX, 31076 Toulouse, France

**Keywords:** *Aspergillus flavus*, Aflatoxin B1, *Aloysia citrodora*, aqueous extract, polyphenols, luteolin-7-diglucuronide

## Abstract

Aflatoxin B1 (AFB1) is a mycotoxin considered a potent carcinogen for humans that contaminates a wide range of crops. Various strategies have been established to reduce or block the synthesis of AFB1 in food and feed. The use of aqueous extracts derived from plants with high antioxidant activity has been a subject of study in recent years due to their efficacy in inhibiting AFB1. In this study, we assessed the effect of *Aloysia citrodora* aqueous extract on *Aspergillus flavus* growth and on AFB1 production. A bio-guided fractionation followed by High Performance Liquid Chromatography (HPLC) and Mass spectrometry analysis of the active fraction were applied to identify the candidate molecules responsible for the dose-effect inhibition of AFB1 synthesis. Our results revealed that polyphenols are the molecules implicated in AFB1 inhibition, achieving almost a total inhibition of the toxin production (99%). We identified luteolin-7-diglucuronide as one of the main constituents in *A. citrodora* extract, and demonstrated that it is able to inhibit, by itself, AFB1 production by 57%. This is the first study demonstrating the anti-Aflatoxin B1 effect of this molecule, while other polyphenols surely intervene in *A. citrodora* anti-AFB1 activity.

## 1. Introduction

Aflatoxin B1 (AFB1) is a mycotoxin produced by different fungal species belonging to the section *Flavi* of the *Aspergillus* genus, *A. flavus* and *A. parasiticus* being the most frequent representatives found as contaminants in food and feed [1,2]. AFB1 has been classified in group I of carcinogenic molecules for humans by the International Agency for Research on Cancer (IARC) [3]; it has also been reported as an immunotoxic and mutagenic agent and is responsible for growth retardation in children [4,5,6]**.** Moreover, AFB1 can also affect other tissues in humans and animals, such as the kidney and liver [7,8] and is considered to be the natural contaminant responsible for the greatest number of DALYs (Death and Disability Adjusted Life Years) [9].

AFB1 is a frequent contaminant of a wide variety of crops in tropical and sub-tropical regions**,** in relation to the temperatures and humidity levels that may favor fungal growth and toxin production [10]. In these regions, around 4.5 billion people are exposed at different levels to AFB1 through their diet [9]. In recent years, global warming has increased the distribution area of AFB1 to countries that were previously considered free. For instance, several studies showed the contamination of different European crops (mainly maize) by this toxin [11,12]. 

Different strategies have been established to limit or block the fungal development and subsequent AFB1 production in crops during pre-harvest, post-harvest, and during storage of food commodities, such as Good Agricultural Practices (GAP) and the use of fungicides. However, nowadays, current concerns about the effect of pesticides on human health and the environment have led to the development of alternative and more sustainable strategies to guarantee food safety. Some natural plant-based compounds have already been proposed to limit or block the synthesis of aflatoxins [13]. For instance, various essential oils as well as some aqueous extracts of plants, herbs, barks, and fruits have been shown to have an antifungal and/or an anti-AFB1 activity [14,15,16,17]. From these studies, it appeared that the active molecules involved in this effect often correspond to phenols, flavonoids, tannins, and terpenes [18,19,20]. Moreover, it has been proposed that there is a correlation between the antioxidant potential of active molecules and their impact on AFB1 inhibition [21,22]. The hypothesis underlying this observation is that AFB1 production could represent a way for the fungus to fight against oxidative stress [22]. The presence of an anti-oxidative agent could therefore indirectly limit the toxin production. 

*Aloysia citrodora* Paláu, also known as *Lemon verbena* or Cedrón, is a shrub belonging to the *Verbenaceae* family that originated in South America. *A. citrodora* is highly used in traditional medicine due to its spasmolytic, cardiotonic, sedative, digestive, and diuretic activities [23,24,25]. Different studies have identified several categories of phytochemicals in *A. citrodora.* For instance, monoterpenes and monoterpenoids such as limonene, geranial, and neral were detected in the essential oil obtained from aerial parts of the plant [26]. In addition, various flavonoids and hydroxycinnamic acids have been identified from methanolic and aqueous extracts [23,24]. 

One previous work already demonstrated the high antioxidative potential of the *A. citrodora* aqueous extract obtained by maceration [14]. This property could be related to the presence of flavonoids such as luteolin and apigenin derivatives, as reported by Portmann et al. [27]. 

Considering the antiradical potential of this plant extract and that AFB1 production could be linked to the oxidative stress level, the present study aims to investigate the antifungal and anti-AFB1 activity of *A. citrodora* aqueous extract. A bio-guided fractionation followed by High Performance Liquid Chromatography (HPLC) and Mass Spectrometry analysis of the active fraction was applied to identify the candidate molecules responsible for the dose-effect inhibition of AFB1 synthesis observed in *Aspergillus flavus*.

## 2. Results and Discussion

### 2.1. Impact of A. citrodora Aqueous Extract on A. flavus Development and AFB1 Production 

#### 2.1.1. Impact on AFB1 Synthesis

**Figure 1:**
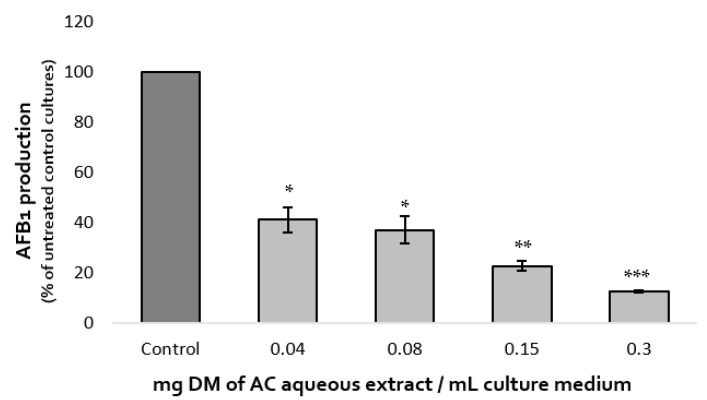
Dose-effect of increasing concentrations of *A. citrodora* aqueous extract on AFB1 production in *A. flavus* NRRL 62477. Results are expressed as the percentage of remaining AFB1 production compared with untreated control cultures ± standard deviation (*n* = 4). ns = no statistically significant change, * *p* value < 0.05; ** *p* value < 0.01; *** *p* value < 0.001.

The effect of increasing concentrations of *A. citrodora* extract on AFB1 production was assessed after 7 days at 27 °C. Dose-effect is shown in Figure 1. AFB1 synthesis was inhibited by 59%, 63%, 77%, and 87% after exposure to 0.04, 0.08, 0.15, and 0.3 mg DM/mL of culture medium, respectively. The IC50_afb1_ (concentration reducing AFB1 production by 50%) of *A. citrodora* extract was 0.05 mg DM/mL culture medium. *A. citrodora* aqueous extract seems to be more efficient against AFB1 than other aqueous extracts already studied, such as aqueous extracts of *Annona muricata*, *Vaccinium myrtillus*, *Micromeria graeca*, for which IC50_afb1_ of 3.3, 1.31, and 0.9 mg DM/mL were respectively reported [12,13,25]. Furthermore, studies conducted on *Mimosa tenuiflora* and *Cannabis sativa* extracts reported them as potential inhibitors of AFB1, with IC50_afb1_ quite similar to those obtained in *A. citrodora* (0.15 mg DM/mL and 0.056 mg DM/mL respectively) [21,28].

#### 2.1.2. Effect on the Fungal Growth and Morphology of *A. flavus*

**Figure 2:**
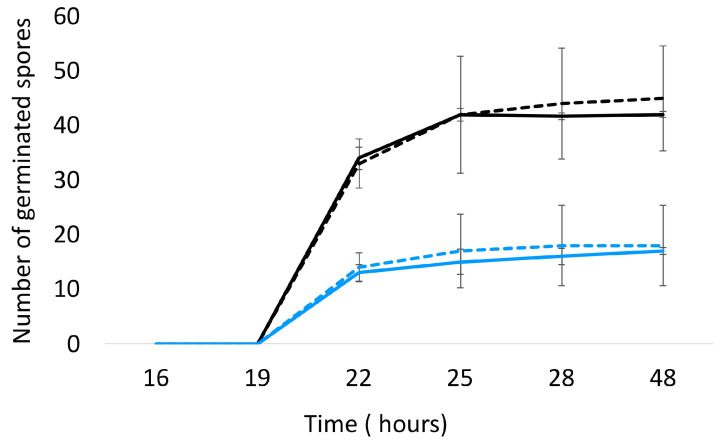
Germination of *A. flavus* NRRL 62,477 spores in the presence (dotted lines) or absence (solid lines) of *A. citrodora* aqueous extract at 0.3 mg DM/mL culture medium. The initial theorical number of spores inoculated onto culture medium was 50 (black) and 25 (blue).

The impact of *A. citrodora* aqueous extract on *A. flavus* development was evaluated at different levels. First, *A. citrodora* aqueous extract at 0.3 mg DM/mL culture medium had no effect on spore germination. As shown in Figure 2, no significant delay in germination or modification of the total number of germinated spores was observed.

Moreover, cultivation of *A. flavus* for 7 days in the presence of *A. citrodora* aqueous extract (0.3 mg DM/mL culture medium) did not significantly modify the growth speed or final diameter of the colony (Table 1). However, a mild increase in total spore number as well as a significant increase in spore density were observed on *A. citrodora* treated colonies after 7 days of incubation. There are only a few data points on the relationship between oxidative stress and fungal sporulation. However, it has been shown that an increase in oxidative stress could decrease sporulation in *Beauveria bassiana* [29]. On the contrary, *Micromaeria graeca* extract was shown to mildly increase spore production in *A. flavus* [15], together with a mean antioxidant capacity [30]. This could be linked with the up-regulation of the *stuA* (stunted) gene, that is involved in conidia development [15,31].

**Figure 3:**
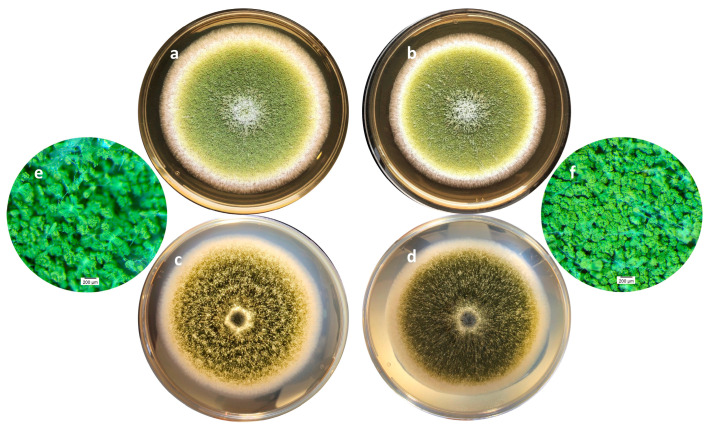
Macroscopic aspects of an *A. flavus* culture after 7 days of incubation at 27 °C in the absence (**a**,**c**,**e**) or presence (**b**,**d**,**f**) of 0.3 mg DM/mL culture medium of *A. citrodora* extract. (**a**,**b**) show the aspect of the colonies under standard lighting; (**c**,**d**) show the aspect of the colonies when lighting under the Petri dish, illustrating the compaction of the basal mycelium in treated culture (**d**). (**e**,**f**) are magnification (×30) of cultures showing the increased spore density in treated culture (**f**) compared with control one (**e**).

On a morphological point of view, cultures with or without of *A. citrodora* aqueous extract displayed a classical general organization of *A. flavus* colonies as well as the usual yellow-green color (Figure 3a,b). However, some differences may be observed, particularly on the surface of the colony, which is finely grainy in controls and appears more plane in treated samples. This corresponds to a greater compaction of the basal mycelium in treated cultures (Figure 3c–f).

**Figure 4:**
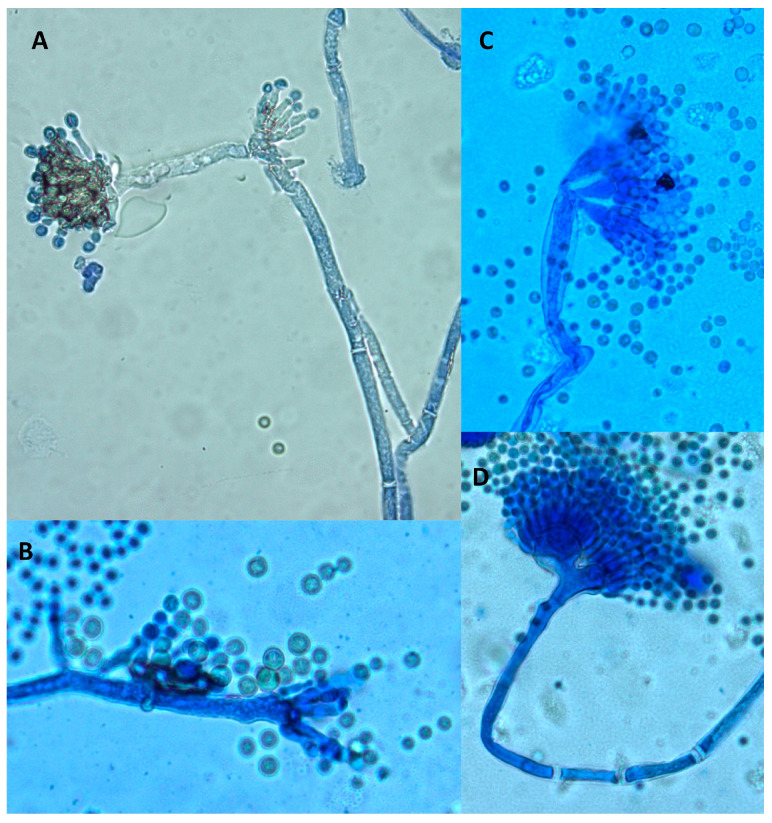
Morphological abnormalities observed in an *A. flavus* culture exposed to 0.3 mg DM/mL culture medium of *A. citrodora* aqueous extract. (**A**,**B**) anarchic phialides on hyphae without vesicles; (**C**,**D**) conidiophores bearing 2 or 3 sporulated vesicles.

A microscopic examination also revealed some differences. Both control and treated cultures displayed classical *A. flavus* features with long, coarse, unbranched conidiophores and radiating biseriate conidial heads. However, in the periphery of treated cultures, some morphological abnormalities were observed (Figure 4). Indeed, some anarchic phialides located directly on hyphae without vesicles or conidiophores displaying two or three sporulated vesicles were observed.

Similar morphological abnormalities were already reported after exposure of *A. flavus* to *Micromeria graeca* extract, together with an inhibition of AFB1 synthesis. It was hypothesized that such abnormalities could be related to the overexpression of *veA* (a gene belonging to the velvet complex) in exposed cultures. Indeed, the impact of *veA* on the morphology of *A. flavus* has been demonstrated [32]. Another hypothesis could be an impact the *nsdC* gene (never in sexual development), whose deletion in *A. flavus* leads to both AFB1 inhibition and the appearance of degenerated conidiophores [33].

### 2.2. Fractionation of A. citrodora Extract and Impact of Fractions on Aflatoxin B1 Production

A previous study reported the high polyphenol content and high antioxidant activity of the aqueous extract of *A. citrodora* [14]. Furthermore, various studies have currently demonstrated that different plant-derived polyphenols are potential inhibitors of AFB1, in relation to their antioxidant properties that may down modulate oxidative stress [21,22]. 

In order to evaluate the r ole of *A. citrodora* polyphenols in AFB1 inhibition, a bio-guided fractionation on a macroporous resin was performed to separate the polyphenols from other compounds. The characterization of the obtained fractions compared withwith *A. citrodora* initial aqueous extract is recapitulated on Table 2.

The characterization of the extract and fractions demonstrated that almost all polyphenols from the initial aqueous extract were concentrated in Fraction 2 (ethanolic). This went with the high antioxidant activity of Fraction 2 (IC50 of 38 mg/L), whereas that of the aqueous fraction (Fraction 1) was below the detection limit of the equipment (>500 mg/L). However, the content of both the initial aqueous extract and fractions in condensed tannins was very limited. This is in agreement with the plant parts used to prepare the extract (leaves). For comparison, aqueous extracts prepared from the bark of plants may be significantly richer in condensed tannins [21].

**Figure 5:**
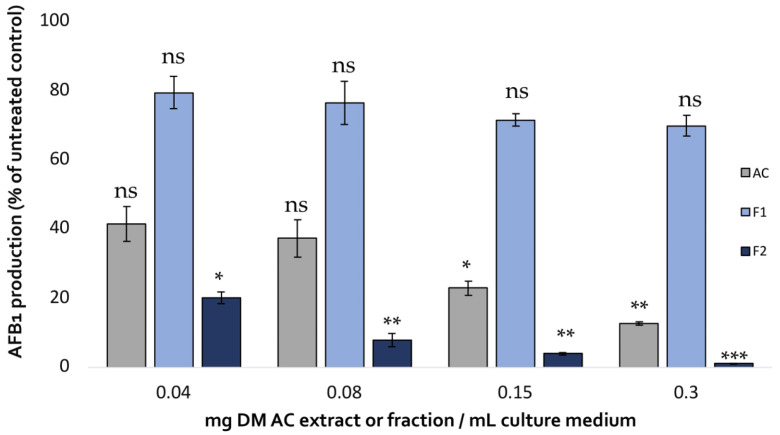
Comparison of the effect of increasing concentrations of *A. citrodora* aqueous extract (grey), Fraction 1 (light blue) and Fraction 2 (dark blue) on AFB1 production in *A. flavus* NRRL 62477. Results are expressed as the percentage of remaining AFB1 production compared with untreated control cultures ± standard deviation (*n* = 4). Statistically significant differences are indicated for the different concentrations of each treatment. ns = no statistically significant change between 2 concentrations, * *p* value < 0.05; ** *p* value < 0.01; *** *p* value < 0.001.

The impact of the two fractions on AFB1 production in *A. flavus* compared with that of the initial aqueous extract is shown in Figure 5.

Fraction 2 showed a dose-dependent AFB1-inhibiting activity that exceeded that of the initial aqueous extract achieving almost total inhibition of the toxin’s production (99% inhibition at 0.3 mg DM/mL culture medium). By contrast, Fraction 1 only led to a limited reduction of AFB1 production, demonstrating the strong correlation between polyphenol content, antioxidative activity, and AFB1 inhibition.

These results are in agreement with several studies demonstrating that plant extracts with significant antioxidant activity, such as *Uncaria tomentosa* (IC50_DPPH_: 13 mg/mL) [14], *Illicium verum* (IC50_DPPH_: 5 mg/mL) [34], and *Curcuma longa* (IC50_DDPH_: 74 mg/mL) [35], may exhibit strong anti-AFB1 activity. This high bioactivity could be related to the presence of antioxidant molecules in the extracts, such as condensed tannins. In fact, the study conducted by Hernandez et al. [21] demonstrated that condensed tannins from the aqueous extract of *M. tenuiflora* are potential inhibitors of AFB1. However, in our extract, the very low content of tannins, particularly in Fraction 2, which is the most active (10 mg/g DM), suggests that some other polyphenolic molecules are responsible for AFB1 inhibition. As an illustration of this hypothesis, Zhou et al. (2015) [36] assessed the efficacy of a tea-derived polyphenol mixture on AFB1 inhibition and demonstrated that gallic acid (part of hydrolysable tannins) and quercetin (flavonoid) were highly effective aflatoxin synthesis inhibitors. 

### 2.3. Identification of the Most Abundant Molecules of the Fraction 2 (Ethanolic)

**Figure 6:**
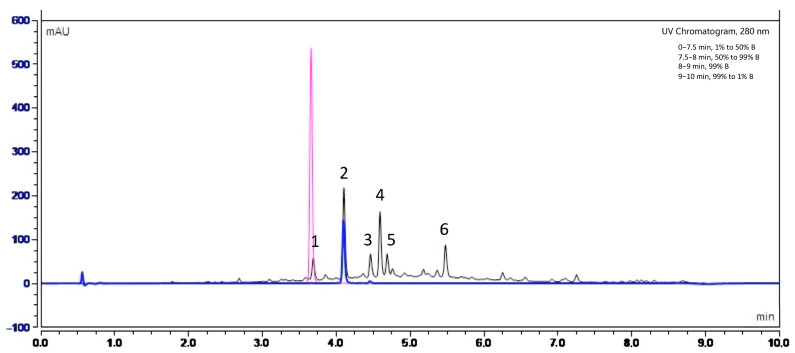
UV chromatogram obtained by high-performance liquid chromatography UV diode-array detection (HPLC-DAD) at 280 nm for Fraction 2 of *A. citrodora* at a concentration of 2 g DM/L (black) and those of commercial standards of luteolin-7-diglucuronide (blue) and caffeic acid (pink). The mobile phase consisted in solvent A (H_2_O−HCOOH, 999:1, *v*/*v*) and B (CH_3_CN−HCOOH, 999:1, *v/v*) and a flow rate of 0.55 mL/min. The identification of compounds corresponding to peaks numbered 1 to 6 are listed in Table 3.

In order to identify the nature of polyphenols present in Fraction 2 of *A. citrodora* extract, the latter was analyzed by high-performance liquid chromatography with UV diode-array detection (HPLC–DAD) and high-performance liquid chromatography coupled with mass spectrometry (HPLC-MS). Figure 6 shows the UV chromatogram of Fraction 2 at 280 nm.

Table 3 details the retention times of each observed peak, the UV-visible bands, and the MS data, including experimental *m*/*z*. Information provided from the literature was taken into account to propose compound identification [37].

Our results suggest that the main molecules present in the extract belong to flavonoids, such as luteolin-7-diglucuronide (peak 2), which is the most abundant compound, followed by chrysoeriol-7-diglucuronide (peak 4) and acacetin-7-diglucuronide (peak 6). A study carried out by Quirantes-Piné et al. (2010) on the characterization of polyphenols in the aqueous extract of *A. citrodora* already reported the presence of luteolin-7-diglucuronide and chrysoeriol-7 diglucurodine [38]. In addition, Sanchez-marzo et al. (2019) reported acacetin-7-diglucuronide as one constituent in the leaves of *A. citrodora* [39]. Glucuronidation of these molecules in plants occurs mainly to enhance their solubility in water [40]. To confirm the presence of luteolin-7-diglucuronide, a pure standard was used. Its superposition with peak 2 is shown in Figure 6.

According to the literature, verbascoside has also been reported as an important constituents of the aqueous extract of *A. citrodora* [41]. However, it was not detected in our extract, despite the use of pure standard. The difference in composition between our extract and those previously reported may be due to the fact that both the type and amount of phytoconstituents in plants are influenced by factors such as their geographical origin, cultivation conditions, harvest seasons and type of soil. Indeed, phenolic compounds are metabolites produced as a defense mechanism against external stress. Thus, their production may directly depend on the environment where the plants are growing [42,43,44]. Finally, we identified the presence of caffeic acid (Figure 6), and possibly apigenin-7-diglucuronide and diosmetin-7-diglucuronide but in smaller proportions, as reported in some *A. citrodora* extracts characterization [39,45].

### 2.4. Effect of Luteolin-7-Diglucuronide on AFB1 Synthesis

Luteolin-7-diglucuronide was found to be the main component of our *A. citrodora* extract. HPLC analysis allowed us to quantify its concentration to 330 µg/mL of extract, which is consistent with the solubility of the molecule in water, set at 388 µg/mL. Thus, we evaluated the impact of that concentration of pure luteolin on AFB1 production. After 7 days of incubation at 27 °C, pure luteolin was able to inhibit by 57% the production of AFB1 in *A. flavus*. This is the first study reporting the impact of luteolin-7-diglucuronide on the production of AFB1. This finding is of great interest since luteolin is a phytoconstituent found in many plants [46]. Therefore, the use of that phenolic compound to inhibit AFB1 contamination of crops could represent an interesting valorization of some by-products of the agro-industry.

Nevertheless, pure luteolin, used at the concentration present in *A. citrodora* extract, was not able to be as efficient in AFB1 inhibition as the aqueous extract. It strongly suggests that other phenolic compounds also intervene, in conjunction with luteolin, to block the toxin’s production. Previous studies have suggested that some polyphenols, such as quercetin [38], gallic acid [47], and p-hydroxybenzoic acids [48], may, for instance, inhibit AFB1 production. A compound of interest could be caffeic acid, which was found in our extract and demonstrated to be able to inhibit AFB1 production [49]. However, concentrations required to observe that effect are much higher than those found in the aqueous extract of *A. citrodora*. Other polyphenols present in *A. citrodora*, such as chrysoeriol-7-diglucuronide or acacetin-7-diglucuronide, could also play a role, but, to date, there is no data available on their possible anti-AFB1 effect. 

Moreover, some studies have demonstrated a possible synergistic effect of the presence of diverse polyphenols, especially regarding their antioxidative properties [50,51]. Such a phenomenon could occur in *A. citrodora* extract, leading to a stronger AFB1 inhibition using the complete aqueous extract compared with that of each pure compound at the same concentration. Such a hypothesis has to be further investigated and could open new perspectives in the control of AFB1 contamination using plant extracts/compounds. 

## 3. Materials and Methods

### 3.1. Chemicals and Reagents 

All analytical solvents and chemicals were purchased from Sigma-Aldrich (St Quentin-Fallavier, France). Luteolin-7-diglucuronide and apigenin-7-glucuronide were purchased from Phytolab (Nantes, France). Ethanol 96% and sodium carbonate were purchased from VWR International (Fontenay sous Bois, France). Ultrapure water was prepared by using Veolia Purelab Classic (Veolia, Toulouse, France). 

### 3.2. Plant Material

*Aloysia citrodora* leaves were bought in a local market in the city of Lima, Peru. Dried plants were ground using a mill equipped with a 2 mm grid, and stored in plastic bottles at 4 °C until use.

### 3.3. Aqueous Extract Preparation 

*A. citrodora* extract was prepared as described by Hernandez et al. [21]. For that, a total of 90 g of ground plant were mixed in 3 L of distilled water and stirred in a magnetic stirrer for 15 h at room temperature. Furthermore, the liquid was centrifugated at 15,000× *g rcf* for 15 min (Sigma 6–16 K Centrifuge, Osterode am Harz, Germany) and filtered through Whatman N° 1 paper. Filtrates were sterilized at 121 °C for 20 min (SMI group UNICOM, Montpellier, France). The final extract was stored at 4 °C until use.

### 3.4. Fractionation of A. citrodora Extract

Microporous Adsorption Resins (MARs) were used to fractionate *A. citrodora* extract. Eighty grams of the adsorbent resin FPX66 (Rohm and Haas, Philadelphia, PA, USA) were preconditioned with ultrapure water (pH 5.5) and packed in a cylindrical glass column (ID × L = 3 × 30 cm) equipped with a fritted disk. 300 mL of the extract at 5.57 g DM/L were loaded into the column. In order to remove sugars, proteins, and other compounds, an elution was first done with 2 BV (bed volume) of ultrapure water, followed by a desorption using 1 BV of 375 mL of 95% ethanol to release compounds retained on the resin, mainly polyphenols. In total, the whole fractionation process was repeated four times, and the aqueous fractions were pooled to obtain Fraction 1 (aqueous), while the four ethanolic fractions were gathered to form Fraction 2 (ethanolic).

### 3.5. Characterization of the Extract and Fractions

#### 3.5.1. Dry Matter Content and Extraction Yield

Dry Matter (DM) content was obtained by weighing each sample before and after drying for 24 h in a Memmert oven at 100 °C (Schwabach, Germany). The extraction yield was calculated using the following equation:

Extraction yield (%) = (weight of extracted plant/weight of plant raw sample) × 100

#### 3.5.2. Determination of the Total Phenolic Content

The total phenolic content was determined using the Folin-Ciocalteu method as described by Hernandez et al. [21]. Twenty µL of the sample were mixed with 10 µL of Folin- Ciocalteu reagent and 170 µL of sodium carbonate at 2.36% in water in a 96 well-microplate. Furthermore, the absorbance at 700 nm was measured using a BMG-Labtech Spectrostar-Nano spectrophotometer (BMG LABTECH SARL, Champigny s/Marne, France) at 45 °C for 45 min. The results are expressed in mg of Gallic Acid Equivalent (GAE) per gram of dry extract.

#### 3.5.3. Determination of the Free Radical Scavenging Activity

DPPH (2,2-diphenyl-1-picrylhydrazyl) free radical was used to assess the antioxidant activity of the extract and fractions [52]. Briefly, seven different concentrations (in a constant volume of 150 µL) were mixed with 150 µL of DPPH solution, and the absorbance was measured at 516 nm after reacting for 40 min in a BMG-LabtechSpectrostar-Nanospectrophotometer (BMG LABTECH SARL, Champigny s/Marne, France). Five replicates for each sample concentration was analyzed. The reaction equation was:

$$f\left(Ce\right)c=1-\frac{A}{A0}$$

where *Ce* is the concentration of the extract (g DM/L), *A* is the absorbance of the extract, and *A*0 is the blank absorbance (DPPH) after 40 min of reaction. The antioxidant activity was determined as the Inhibitory Concentration 50 (IC50), which corresponds to the concentration of the extract that reduces 50% of the DPPH radicals. Results are expressed in milligrams of extract per liter (mg/L).

*IC*50 is calculated by using the following linear regression:

$$IC50=0.5-\frac{a}{b}$$

where *a* is the origin ordinate, and *b* is the slope.

#### 3.5.4. Determination of the Condensed Tannins Content

The condensed tannins content of each extract was quantified by an adaptation of Waterman and Mole [53]. Briefly, samples were diluted with distilled water to obtain, after hydrolysis, an absorbance equal to or lower than 0.200. For each sample, two tubes were used, 1 mL of distilled water was added in 2 mL of the diluted sample, and 3 mL of concentrated hydrochloric acid (12 N) were added. The sample tubes were incubated at 100 °C for 30 min, while the control tubes were immersed in crushed ice. The heated tube was then collected and cooled on crushed ice. Finally, 0.5 mL of ethanol was added to all tubes, and they were vortexed for 10 s. The absorbance was assessed at 550 nm using a Shimadzu UV 1800 spectrophotometer (Shimadzu Corp, Kyota, Japan). The tannin concentration was calculated using the following equation:

Tannin concentration (mg/g) = [0.3866 × (sample absorbance − control absorbance) × dilution factor]/dry matter

The test was done in triplicate and the results are expressed in mg of condensed tannins per g of dry matter (DM) ± standard deviation (SD).

### 3.6. Characterization of the Bioactive Compounds Present in A. citrodora Extract and Its Fractions

#### 3.6.1. HPLC-DAD Analysis

The characterization of the bioactive molecules was done as described by Quirantes- Piné et al. [37]. Analyses were carried out on an Ultimate 3000 UPLC (Thermo-Fisher, Illkirch-Graffenstaden, France), including a diode array detector (DAD). An EvoC18 column (2.6 µm, 150 × 2.1 mm, Phenomenex, Le Pecq, France) was used for all assays. The mobile phases consisted of solvents A and B, which were respectively water: acetonitrile (90:10 *v*/*v*) with 1% of formic acid and acetonitrile. The linear gradient applied was 5% B 0–25 min, 20% B 25–30 min, 40% B 30–35 min, 5% B 35–40 min. The separation was performed at 30 °C with a flow rate of 0.5 mL/min. The injection volume of each sample was 20 µL. The UV detection was made in the λ range of 190–450 nm. 

To better identify some of the components of the *A. citrodora* extract, the following standards were used: luteolin-7-diglucuronide, verbascoside, apigenin-7-glucuronide and caffeic acid. Standards were dissolved in Dimethyl sulfoxide (DMSO) to obtain a final concentration of 700 mg/L. Different concentrations of the standards were injected (50–100–200–300–400–500 mg/L) in order to draw calibration curves.

#### 3.6.2. Mass Spectrometry Analysis

The liquid chromatography system consisted of an Ultimate 3000 ultrahigh-performance liquid chromatography (UHPLC) equipped with a photodiode array detector (DAD, Thermo Scientific, Waltham, MA, USA). The column used was an Acquity UPLC HSS T3, 2.1 × 100 mm, 1.8 µm (Waters, Milford, CT, USA), which contained a trifunctional C18 end-capped phase. The flow rate was 0.55 mL min^−1^ and the gradient conditions were as follows: solvent A (H_2_O–HCOOH, 999:1, *v/v*), solvent B (CH_3_CN–HCOOH, 999:1, *v/v*); 0–7.5 min, 1% to 50% B (linear gradient); 7.5–8 min, 50% to 99% B (linear); 8–9 min, 99% B (isocratic); and 9–10 min, 99% to 1% B (linear). The Ultimate 3000 UHPLC system was coupled online with an ISQ EM simple quadrupole mass spectrometer (Thermo Scientific), with heated electrospray ionization operating in both positive- and negative-ion mode. In the source, the sheath gas pressure was 53.4 psi, the auxiliary gas pressure was 6.1 psi and the sweep gas pressure was 0.1 psi. The vaporizer temperature was set at 310 °C, and the ion transfer tube temperature was set at 300 °C. The capillary voltage was set at 3 kV in positive-ion mode and −2 kV in negative-ion mode. The mass spectra were acquired in full scan mode over an *m*/*z* range of 110–1600. The speed of mass spectrum acquisition was set at 7450 *m*/*z* s^−1^.

### 3.7. Effect of A. citrodora Extract and Its Fractions on Aspergillus flavus and AFB1 Synthesis

#### 3.7.1. Fungal Strain and Culture Conditions

For all experiments, *A. flavus* strain NRRL 62,477 was used [54]. Briefly, a suspension of spores was prepared in Tween 80 from a seven-day culture on Malt Extract Agar (MEA) (Biokar Diagnostics, Allone, France), and its concentration was adjusted to 10^5^ spores/mL after counting on Malassez cells. Ten µL of spore suspension (1000 spores) were inoculated centrally onto culture medium made of 18 mL of MEA added with 2 mL of autoclaved *A. citrodora* extracts or fractions prepared at four different concentrations (0.04, 0.08, 0.15, and 0.3 mg DM/mL of culture medium) by dilution in water. Control cultures were made by adding 2 mL of distilled water in MEA. For luteolin-7-diglucuronide assays, the standard was dissolved in water/ethanol (1:1 *v*:*v*) to obtain a final concentration of 220 mg/L. Three ml of this solution were mixed with 17 mL of MEA to reach a final concentration of luteolin of 33 µg/mL culture medium. Control cultures were obtained by adding 3 mL of distilled water/ethanol (1:1) to 17 mL of MEA. All cultures were then incubated for 7 days at 27 °C in the dark before analysis.

#### 3.7.2. Impact of *A. citrodora* Aqueous Extract on *A. flavus* NRRL 62,477 Development and Morphology

The impact of *A. citrodora* on fungal development in *A. flavus* was conducted as previously described [15]. All examinations and measurements were done in triplicate.

##### Effect on Fungal Growth

Final growth was estimated by the measurement of the culture’s opposite diameters after 7 days of culture. For mycelium dry weight measurement, culture medium was layered with 8.5 cm sterile cellophane film disks (Hutchinson, Chalette-Sur-Loing, France) before inoculation. At the end of the incubation period, cellophane disks were removed from the medium, placed to dry at 60 °C for 48 h and weighted on an analytical balance. To determine the fungus final dry weight, the mean weight of three desiccated control cellophane disks was subtracted. 

##### Effect on Spore Germination and Production

To assess the impact of *A. citrodora* extract on spore germination, a theoretical total number of 25 and 50 spores were inoculated on MEA supplemented (treated) or not (control) with 0.3 mg DM/mL of culture medium. The appearance of germination tubes was monitored under a binocular stereomicroscope (Olympus, Rungis, France) at 19, 22, 25, 28, and 48 h. The total number of germinated spores was counted after 5 days of culture.

To measure if the total number of spores was impacted by the extract, 7-days-old colonies grown with or without the *A. citrodora* extract at 0.3 mg DM/mL of culture medium were gently washed with 3 × 40 mL Tween (0.05%), and spores were removed by scraping off the culture. Spore suspension was homogenized, dilutions were prepared, and spores were counted on Malassez cells to determine the total spore count (SC). Spore Density (SDe) was calculated with the following formula:

SDe = SC/(π*r*^2^)

where *r* is the mean colony radius.

##### Effect on Fungal Morphology 

The impact of *A. citrodora* aqueous extract on the morphological features of *A. flavus* NRRL62477 was characterized after 7 days of culture at 27 °C. Macroscopic aspects of colonies (color, thallus margin and texture, aspect of conidial heads, colony reverse) were observed under stereomicroscope (×6 to ×60 magnifications). Microscopic features (shape of vesicle, conidiophore, number and organization of sterigmata, shape of conidia…) were examined under an optical microscope (Olympus, Rungis, France) at ×400 and ×1000 magnifications.

#### 3.7.3. Aflatoxin Extraction and HPLC Quantification

AFB1 extraction and quantification were done as previously described [15]. Culture media were mixed with 30 mL of HPLC-grade absolute chloroform and shaken at 200 rpm for 1 h at room temperature. Supernatants were filtered through a Whatman 1PS phase separator (GE Healthcare Life Sciences, Vélizy-Villacoublay, France). Two mL of the filtrates were evaporated to dryness in a STUART SBH200D/3 sample concentrator (Stuart equipment, Paris, France) at 45 °C and redissolved in 2 mL of acetonitrile. Furthermore, they were filtered through 0.45 μm PTFE disk filters (Thermo Fisher Scientific, Illkirch-Graffenstaden, France) and placed in HPLC vials. AFB1 quantification was done using an Ultimate 3000 UPLC (Thermo-Fisher Scientific, Illkirch-Graffenstaden, France) with an EvoC18 column (3 µm, 150 × 3.2 mm, Phenomenex, Le Pecq, France) conditioned at 27 °C. In order to separate AFB1, an elution program consisting of an isocratic mixture of acetonitrile and water (25:75 *v:v*) was used. The mobile phase had a flow rate of 1.2 mL/min and 10 µL of the sample was injected. Aflatoxin B1 was detected by using a fluorescent detector at 365 (430) nm excitation (emission) wavelengths. A diode matrix detector (DAD) coupled to the system was used to analyze the UV spectra. AFB1 levels were calculated based on a standard calibration curve in a range of 0.16 to 20 µg/mL.

### 3.8. Statistics

A Student *t*-test was used to analyze the differences between control and treated samples. Differences were considered statistically significant with *p* values lower than 0.05. Data analysis was done using R studio Software (version 1.4.1717). Graphical values are represented as the mean ± standard deviation (SD) of four different assays.

## 4. Conclusions

The present study demonstrates the potent inhibition of AFB1 production by *A. citrodora* aqueous extract without impact on fungal growth. A bio-guided fractionation demonstrated that this inhibition is mainly related to the presence of polyphenols displaying high antioxidant activity. Among the present compounds, luteolin-7-diglucuronide was the most abundant, and it was found to be responsible of more than half of the observed effect. Such a finding could open the door to an interesting valorization of some agro-industrial by-products since many of them could contain luteolin. Our results also suggest that AFB1 inhibition due to the aqueous extract is the result of an additive or synergistic effect of different phenolic compounds. The higher efficacy of the complete aqueous extract shows that a simple solvent-free extraction procedure of common abundant natural resources, with no further purification step, could represent a sustainable and eco-friendly alternative to pesticides in the fight against AFB1 contamination of crops. 

## Figures and Tables

**Table 1:** Effect of *A. citrodora* extract (0.3 mg DM/mL of culture medium) on the development of *A. flavus* NRRL 62477.

	Fungal Growth			Sporulation	
	Growth Speed (mm/d) ^1^	Colony Final Diameter (mm)	Colony Dry Weight (mg)	Spore Quantity (×10^8^)	Spore Density ^2^ (×10^6^)
Control	5.3 ± 0.02 ^a^	74.8 ± 0.3 ^a^	115.33 ± 8 ^a^	33.7 ± 2.08 ^a^	76.6 ± 4.43 ^a^
Treated ^3^	5.2 ± 0.05 ^a^	73.2 ± 0.8 ^a^	122 ± 7 ^a^	37.2 ± 2.53 ^a^	88.4 ± 5.22 ^b^

^1^: mm/d: milimeter per day, ^2^: quantity of spores per cm^2^ of fungal colony, ^3^: *A. flavus* cultivated for 7 days in the presence of *A. citrodora* extract at 0.3 mg DM/mL culture medium. For each parameter, significant differences between control and treated cultures are indicated with different letters (*p* value < 0.05).

**Table 2:** Characterization of *A.citrodora* aqueous extract and its aqueous (Fraction 1) and ethanolic (Fraction 2) fractions after separation on macroporous resin.

Fractionated Extracts	Concentration (g DM/L)	Total Polyphenol Content (mg GAE/g DM Extract)	Condensed Tannins (mg/g DM)	Anti-Oxidant Activity Measured by DPPH Assay (IC50 * in mg/L)
*A. citrodora* aqueous extract	6.1	220 ± 8	8.0 ± 1.2	58
Fraction 1 (Aqueous)	1.0	35 ± 1	1.0 ± 0.5	>500
Fraction 2 (Ethanolic)	1.2	395 ± 7	10.0 ± 0.8	38

DM: Dry Matter; GAE: Gallic Acid Equivalent; DPPH: 2,2 Diphenyl 1 pycrilhydrazyle. * Concentration of extract or fraction reducing 50% of DPPH.

**Table 3:** MS and UV-visible bands for the main possible compounds in Fraction 2.

	Retention Time (min)	*m*/*z* Experimental	Molecular Formula	λ Max (nm)	Proposed Compounds
1	3.65	179	C_9_H_8_O_4_	298 sh, 323	caffeic acid
2	4.09	637	C_27_H_25_O_18_	255, 267 sh, 348	luteolin-7-diglucuronide
3	4.45	621	C_27_H_25_O_17_	267, 335	apigenin-7-diglucuronide
4	4.59	651	C_28_H_27_O_18_	252, 267 sh, 345	chrysoeriol-7-diglucuronide
5	4.69	651	C_28_H_27_O_18_	250 sh, 268,336	diosmetin-7-diglucuronide
6	5.47	635	C_23_H_22_O_13_	252 sh, 272,342	acacetin-7-diglucuronide

Note: sh, shoulder.

## Data Availability

Data will be available on request to corresponding author.
